# The Effect of EVA and TPU Custom Foot Orthoses on Running Economy, Running Mechanics, and Comfort

**DOI:** 10.3389/fspor.2019.00034

**Published:** 2019-09-19

**Authors:** Ken Van Alsenoy, Joong Hyun Ryu, Olivier Girard

**Affiliations:** ^1^Exercise and Sport Science Department, ASPETAR Orthopedic and Sports Medicine Hospital, Doha, Qatar; ^2^Centre for Health, Activity and Rehabilitation Research (CHEAR), Queen Margaret University, Musselburgh, United Kingdom; ^3^Department of Sport Sciences, ASPIRE Academy for Sports Excellence, Doha, Qatar; ^4^Murdoch Applied Sports Science (MASS) Laboratory, Murdoch University, Perth, WA, Australia; ^5^Athlete Health and Performance Research Center, ASPETAR Orthopedic and Sports Medicine Hospital, Doha, Qatar

**Keywords:** orthotics, material resilience, economy of locomotion, gait, running, kinetics

## Abstract

Custom made foot orthoses (CFO) with specific material properties have the potential to alter ground reaction forces but their effect on running mechanics and comfort remains to be investigated. We determined if CFO manufactured from ethyl-vinyl acetate (EVA) and expanded thermoplastic polyurethane (TPU) materials, both compared to standardized footwear (CON), improve running economy (RE), running mechanics, and comfort at two running speeds. Eighteen well-trained, male athletes ran on an instrumented treadmill for 6 min at high (HS) and low (LS) speeds corresponding to and 15% lower than their first ventilatory threshold (13.8 ± 1.1 and 11.7 ± 0.9 km.h^−1^, respectively) in three footwear conditions (CON, EVA, and TPU). RE, running mechanics and comfort were determined. Albeit not reaching statistical significance (*P* = 0.11, η^2^ = 0.12), RE on average improved in EVA (+2.1 ± 4.8 and +2.9 ± 4.9%) and TPU (+0.9 ± 5.9 and +0.9 ± 5.3%) compared to CON at LS and HS, respectively. Braking force was decreased by 3.4 ± 9.1% at LS and by 2.7 ± 9.8% at HS for EVA compared to CON (*P* = 0.03, η^2^ = 0.20). TPU increased propulsive loading rate by 20.2 ± 24 and 16.4 ± 23.1% for LS and HS, respectively compared to CON (*P* = 0.01, η^2^ = 0.25). Both arch height (*P* = 0.06, η^2^ = 0.19) and medio-lateral control (*P* = 0.06, η^2^ = 0.16) showed a trend toward improved comfort for EVA and TPU vs. CON. Compared to shoes only, mainly EVA tended to improve RE and comfort at submaximal running speeds. Specific CFO-related running mechanical adjustments included a reduced braking impulse occurring in the first 25% of contact time with EVA, whereas wearing TPU increased propulsive loading rate.

## Introduction

Custom foot orthoses (CFO) are increasingly used to alter the magnitude and resultant direction of ground reaction forces (GRF) under the foot by modifying foot-surface interaction. In previously injured athletes, for instance after suffering a lateral ankle sprain, wearing CFO decreased the inversion moment around the subtalar joint axis (Kirby, 2017). The magnitude of the reduction in subtalar joint inversion moment, ultimately altering GRF production during locomotion, directly depends on the amount of customization by molding and/or posting (McCormick et al., 2013). This individualized surface geometry, in combination with materials used, also likely determine comfort, cushioning, and bending stiffness (Mundermann et al., 2003a,b; Kirby and Werd, 2014).

Running Economy (RE) is one important factor, in addition to maximal oxygen uptake (VO_2max_) and the fraction of VO_2max_ that can be sustained, which determines exercise capacity (Karp, 2008; Shaw et al., 2013). Efficiency of elastic return through foot-surface interaction and the amount of GRFs produced are important modifiable biomechanical factors determining RE (Arellano and Kram, 2014; Barnes and Kilding, 2015; Moore, 2016). There is conflicting evidence in the literature regarding the effect CFO have on RE. Higher VO_2_ values were reported in runners with a history of running related injuries both at ~14 km.h^−1^ and ~16 km.h^−1^ when using CFO compared to control (i.e., without CFO), while flexible and semi-rigid CFO had similar effects (Hayes et al., 1983). Contrastingly, RE on average improved by ~8% when male endurance trained runners ran at five preset speeds ranging ~11–15 km.h^−1^, while wearing CFO compared to a shoe fitted support (Burke and Papuga, 2012). In these aforementioned studies, however, stride pattern was not characterized to offer a possible biomechanical explanation for CFO-related adjustments in RE. Additionally, previous literature often lacks reporting the precise nature of customization (e.g., type of negative foot model, amount and area of molding and/or posting, materials used, weight of different footwear conditions), which limits strength of comparison between available studies (Fuller et al., 2015). The use of different running shoes between runners (Burke and Papuga, 2012) and set (i.e., absolute) running speeds for a range of runners with rather different levels of overall aerobic fitness (Hayes et al., 1983; Burke and Papuga, 2012) also participate to increase interindividual variability in the response to a CFO intervention.

CFO might have the potential to improve RE through their shape and material characteristics. It is likely (but unknown) that more favorable running mechanics could be attributed to these surface characteristics. A modified stride mechanical pattern, for instance by decreasing vertical impact forces, peak medial–lateral force and horizontal braking GRF and/or by increasing horizontal propulsive GRF, has the potential to alter RE (Moore, 2016). Compared to running in sport shoes only, MacLean et al. (2008) observed that the addition of CFO decreases vertical impact peak force (~6%) and vertical loading rate (~12%), yet the effect on RE was not reported in this study. While the effects of CFO on vertical GRF are relatively well-described, there is comparatively less information on the impact this may have on the horizontal GRF component and its relationship with RE. Chang and Kram (1999) identified that, for well-trained recreational runners running at ~12 km.h^−1^, the metabolic cost for generating horizontal forces is about four times higher than for vertical forces. This suggests that the collection and analysis of horizontal GRF should be considered when evaluating the effects CFO on RE.

Materials used to produce CFO vary widely. Flexible shank-dependent CFO are commonly made of EVA or polyurethane foams. According to Zeintl (2018), EVA has a 37% resilience elasticity using a standard ball rebound lab test. Compared to EVA, expanded thermoplastic polyurethane (TPU) (Infinergy®, BASF, Germany) achieves a rebound of 55%. When used in the midsole of a running shoe, TPU was associated with a ~4% improvement in RE when compared to running with conventional running shoes (Sinclair et al., 2016). Even though no reporting was made on running mechanics in this study, Worobets et al. (2014) mechanically tested the energy loss using actuated compression testing and found hysteresis values of 31.3–32.3 and 20.9–22.3% for EVA and TPU midsole shoes, respectively. This suggests, but is yet to be verified, that increases in resilience might lie at the basis of its positive effect on RE.

Material characteristics (i.e., density and stiffness) can strongly influence the perception of comfort and is key when deciding to keep wearing CFO or not. Comfort is defined by individual preference and is in turn influenced by many factors such as perception of pain, fatigue, and possibly running speed (Mundermann et al., 2003b). Early comfort studies showed that single verbal ratings (Milani et al., 1995) or even a single five point Likert scale (Hennig et al., 1996) proved not sensitive and provided unreliable results. An assessment tool for measuring comfort, focusing on a range of perceptions (e.g., cushioning, amount of movement control) linked to specific foot sections (e.g., heel, midfoot, and forefoot of footwear and orthotics) was subsequently developed (Mundermann et al., 2002). Different items from this tool (overall comfort, heel cushioning and heel cup fit and medial-lateral control) correlated with improved RE (Burke and Papuga, 2012) when wearing CFO. However, the effect of specifically wearing EVA or TPU materials on comfort and how this relates to alterations in running mechanics and RE at different speeds is unknown.

The aim of this study was to determine the effect of CFO manufactured from EVA and TPU materials (identical shape but different resilience and stiffness characteristics), both compared to a control condition (shoes only), on measures of RE, comfort for different perceptions and locations under the foot (heel, medial arch and forefoot) and running mechanics with special reference to horizontal force production (e.g., braking and propulsive forces) when running at two individualized submaximal speeds. We first hypothesized that, compared to control, EVA and TPU materials would improve RE and increase comfort (for cushioning and control, in general and under the heel, arch and forefoot), due to more efficient running mechanics (e.g., lower vertical impact forces and loading rates, less mechanically-demanding forward-orientated forces). We further hypothesized that the magnitude of these changes will be larger while wearing CFO made of TPU compared to EVA due to the higher resilience material properties.

## Methods

### Participants

Twenty-one male well-trained athletes (mean ± SD age, 38.9 ± 5.1 years; stature, 175.3 ± 5.8 cm; body weight, 74.9 ± 7.7 kg) were recruited for this study. They trained (running and swimming and/or cycling) on average 8.8 ± 3.7 h per week in the 3 months leading up to the data collection with an average weekly running distance of 37.6 ± 26.7 km. During training, participants spent on average 3.8 ± 2.6 h in low intensity, 2.7 ± 1.7 h in medium intensity, 2.2 ± 0.8 h in a high-intensity workout, with also 1.9 ± 1.3 h dedicated to resistance training. Three participants dropped out of the study, one for personal reasons, the second because he couldn't complete the full protocol, the third due to illness. In our final sample of eighteen participants, thirteen were rearfoot strikers, one was a midfoot striker and four were forefoot strikers at 10 km.h^−1^. Two separate raters (KVA and OG) agreed on foot strike pattern, using sagittal plane video-analysis at the level of the foot at a sampling frequency of 240 Hz using an iPhone 6 (Apple, California, US). The participants had a foot structure of median (min, max) 7 (−6, +11) for the left and right foot, as determined by the Foot Posture Index FPI-6 that was scored after completing the last session (KVA). Reference values were labeled as normal (0 to +5), pronated (+6 to +9), highly pronated (10+), supinated (−1 to −4) and highly supinated (−5 to −12) (Redmond et al., 2006). Participants had no known history of cardiovascular, neurological, or orthopedic problems, were injury free for the 3 months leading up to the data collection and gave written informed consent prior to participation in the study. Ethical approval for the study was provided by the Anti-Doping Laboratory Ethics Committee in Qatar (IRB Application Number 2017000201) and was undertaken according to the principles outlined in the Declaration of Helsinki.

### Experimental Protocol

Participants attended the lab on four separate occasions. The first visit aimed at determining the individual ventilatory threshold and corresponding running speed that was used for the three following intervention sessions. The remaining three visits consisted of running at two sub-maximal speeds in different footwear conditions. The second visit was the control session where participants ran with standardized (i.e., only shoe liner inserted) footwear (CON). During the third and fourth session CFO (EVA and TPU) were inserted bilaterally in participants shoes, before the warm-up and for the rest of the session, with the order of intervention randomized between sessions. Participants were asked to avoid strenuous exercise in the 12 h, as well as refrain from food and caffeine for 4 h preceding their visits to the laboratory and were encouraged to replicate their diet and training pattern for all visits. Laboratory conditions were similar throughout all running sessions (mean ± SD temperature 20.7 ± 0.2°C, relative humidity 60.4 ± 0.6%). Time of day was standardized for each participant over all sessions. The participants and the researcher who was directly involved in guiding the runners during the running protocol were visually blinded from the CFO materials.

### Running Bouts

#### Incremental Test (Visit 1)

Each participant completed a continuous, maximal incremental running test. Briefly, participants started running at 9 km.h^−1^ with speed increases of 0.5 km.h^−1^ every 30 s. The test ended with voluntary exhaustion of the participants. Verbal encouragement was only given by the researcher guiding the runners throughout the session. Ventilatory threshold was determined using the criteria of an increase in minute ventilation (V_E_)/Oxygen uptake (VO_2_) with no increase in V_E_/Carbon dioxide (VCO_2_) and the departure from linearity of V_E_ (Davis, 1985).

#### Sub-maximal Runs (Visits 2, 3, and 4)

After a 10 min warm-up at 10 km.h^−1^, followed by a 3 min break used to put on the mask to collect expired gases, participants ran two, 6-min trials: One at an intensity corresponding to the speed associated with the first ventilatory threshold (HS or high speed) and one at a speed 15% below the first ventilatory threshold (LS or low speed), with 3 min recovery in between. The order in which LS and HS conditions were applied was randomized across participants, but held constant for each individual throughout their sessions. The complete timing sequence from warm-up to finish was strictly controlled and guided by visual and verbal cues.

### Footwear

**Figure 1:**
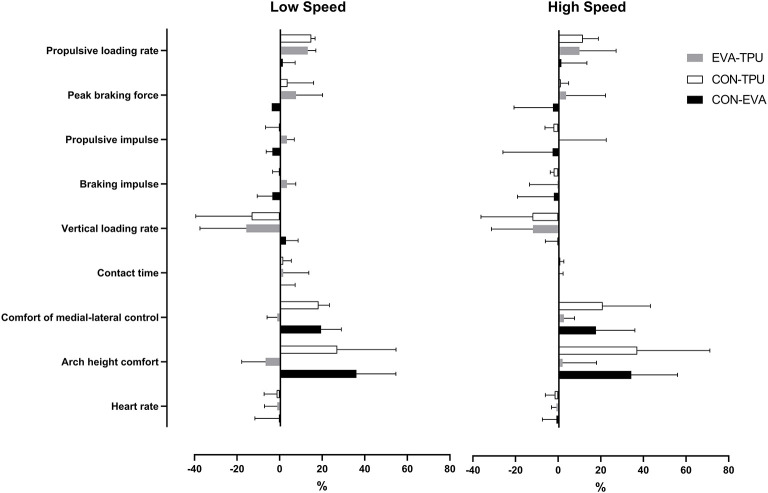
**Left:** a pair of original liners (CON) of shoes, **Middle:** a pair of the custom Thermoplastic Poly-Urethane orthoses (TPU) and **Right:** a pair of custom Ethyl-Vinyl Acetate orthoses (EVA).

During all running the participants used neutral like running shoes (Pearl Izumi N2v2, Colorado, US) with an average European shoesize of 43.6 ± 1.6. At the end of the second visit (control session using shoes with its original shoe liner), each participant received two pairs of CFO based on an individual non-weight bearing 3D scan of the foot using a Delcam iCube scanner (Elinvision, Karmelava, Lithuania) completed at the end of the first visit. CFO were designed by an experienced sport podiatrist with nearly 20 years of experience, using the Orthomodel Pro CAD software (Autodesk, California, USA). Briefly, scans were imported into the software, markers were placed over the heel, first- and fifth metatarsal and medial arch. A base model surface was adjusted to match the contour of the foot using cross-sectional views from the heel to the forefoot. The thickness of the orthotic was arbitrary set to 8 mm in an attempt to maximize the potential of the TPU beats inside the Infinergy® material (BASF, Ludwigshafen, Germany). All CFO were direct-milled out of EVA and TPU and manually finished to fit inside the shoes (Figure 1).

On initial fitting and again before the start of the third session (shoes with the first pair of CFO), participants were asked if the CFO were comfortable and if any adjustments were necessary. When adjustments were made (4 out of 18 participants), they were done on both pairs of CFO to keep an identical shape. No additional adjustment in shape were made between the third and fourth sessions.

Wear-in time between the first and second intervention session was 4.5 ± 2.5 and 4.6 ± 2.8 days between the second and last intervention session. The weight of the three footwear conditions was on average 600.3 ± 32.0, 647.3 ± 36.0, and 681.1 ± 35.7 g for the shoes with its original liners (CON), with the custom EVA orthoses (EVA) and with the custom TPU orthoses (TPU), respectively.

### Data Measure

#### Metabolic Card

A Jeager™ Oxycon Mobile cardio pulmonary exercise testing unit (Carefusion, Hoechberg, Germany) was used to record breath-by-breath and cardio-respiratory data. Prior to each session, calibration of gas sensor was completed for ambient air and a known gas mixture (16% O_2_, 5% CO_2_). Turbine was calibrated using a 3-Liter (±0.4%) syringe and automated High and Low flow ventilation. The metabolic cart was suspended from the ceiling next to participants, so they didn't have to support the additional weight of the system when running.

#### Instrumented Treadmill

An instrumented treadmill (ADAL3D-WR, Medical Developpement - HEF Tecmachine, France) was used for all running conditions (incremental test, constant speed running). Briefly, it is mounted on a highly rigid metal frame, set at 0°grade incline, fixed to the ground through four piezoelectric force transducers (KI 9077b; Kistler, Winterthur, Switzerland) and installed on a specially engineered concrete slab to ensure maximal rigidity of the supporting ground (Girard et al., 2017).

### Data Analysis

#### Cardiorespiratory and Metabolic Variables

Breath-by-breath gas samples were first averaged every 15 s and subsequently expressed as the average of the last 2 min of each 6-min run. Oxygen uptake expressed in both absolute (VO_2_ in mL.min^−1^) and relative (RVO_2_ in mL.kg^−1^.min^−1^) terms, V_E_ (L.min^−1^), breathing frequency (BF) (breaths.min^−1^), tidal volume (VT) (L) were determined. Heart rate (HR) (beats.min^−1^) was continuously measured by short-range telemetry (Polar, Kempele, Finland). Running Economy (RE) was calculated as the VO_2_ per bodyweight over speed, expressed in milliliters of oxygen consumed per kilogram per kilometer (mL.kg^−1^.km^−1^).

#### Kinetic Variables

Over the last 2 min of each 6-min run, three-dimensional ground reaction force was continuously sampled at 1,000 Hz. Ten continuous steps recorded at 4 min 15 s, 4 min 45 s, 5 min 15 s, and 5 min 45 s were subsequently averaged for final analysis. After appropriate filtering (Butterworth-type 30 Hz lowpass filter), data were averaged over the support phase of each step (vertical force above 30 N). Further main spatio-temporal variables: contact time (s), flight time (s), step frequency (Hz) were reported. Vertical stiffness (K_vert_ in kN·m^−1^) was calculated as the ratio of peak vertical forces (F_zmax_ in N) to the maximal vertical downward displacement of center of mass (Δz in m), which was determined by double integration of vertical acceleration of center of mass over time during ground contact (Cavagna, 1975; Morin et al., 2005). Leg stiffness (K_leg_ in kN·m^−1^) was calculated as the ratio of F_zmax_ to the maximum leg spring compression (ΔL) (Δz + L0 - √L0^2^ – [0.5 × running speed × contact time]^2^, in m), both occurring at mid-stance (Morin et al., 2005). Initial leg length (L0, great trochanter to ground distance in a standing position) was determined from participant's stature as L0 = 0.53 × stature (Morin et al., 2005). Finally, vertical mean/peak loading rate was calculated as the mean/peak value of the time-derivate of vertical force signal within the first 50 ms of the support phase, and expressed in N·s^−1^(De Wit et al., 2000).

Also, horizontal forces were analyzed with main variables defined as: peak braking and peak propulsive forces (BW) and the timing (ms) when these events occurred from initial contact; the duration of braking and propulsion forces (ms); the braking and push-off impulse (m.s^−1^) and instantaneous loading rates (N.s^−1^).

#### RPE and Comfort Measures

Rating of perceived exertion (RPE) was measured every 30 s during the continuous incremental test and the two steady-state runs using the 6-20 Borg scale (Borg, 1982). Within the first minute after finishing HS and LS runs, a global (6-min run) RPE value was collected.

A modified version of the footwear comfort assessment tool, developed and tested on reliability by Mundermann et al. (2002), was used to assess comfort associated with wearing each footwear condition using an iPad mini (Apple, California, US). This scale was used in previous studies to assess footwear comfort (McPoil et al., 2011; Burke and Papuga, 2012). For this study, only six of the nine items (“overall comfort,” “heel cushioning,” “forefoot cushioning,” “medio-lateral control,” “arch height,” and “heel cup fit”) were scored on a digital, 150 mm VAS scale where 0 was defined as “not comfortable at all” and 150 “most comfortable condition imaginable.”

### Statistical Analysis

All physiological, mechanical and perceptual dependent variables collected while running in the three footwear conditions over two speeds (HS, LS) were compared using a two-way ANOVA with repeated measures [Condition (CON, EVA, TPU) × Speed (LS, HS)] after confirming a normal distribution (Shaphiro-Wilk), homogeneity (Levene's), and sphericity (Mauchley's). A Greenhouse-Geisser correction was performed to adjust the degree of freedom if an assumption was violated, while a Šídák *post hoc* multiple comparison was performed if a significant main effect was observed for condition and an LSD *post hoc* comparison for speed. Partial eta-squared were calculated as a measure of effect size, with values of 0.01, 0.06, and >0.14 considered as small, medium and large, respectively (Cohen, 1988). The level of significance was set at *P* ≤ 0.05. All statistical analyses were performed in IBM® SPSS® Statistics for Windows v.24 (IBM Corp., Armonk, NY, US).

## Results

The incremental test lasted on average 9.3 ± 1.6 min and participants ventilatory threshold was reached at a running speed of 13.8 ± 1.1 km.h^−1^ (corresponding to 73.5 ± 3.3% of the maximal reached speed) and labeled HS. The LS was 15% slower corresponding to an average running speed of 11.7 ± 0.9 km.h^−1^.

**Figure 2:**
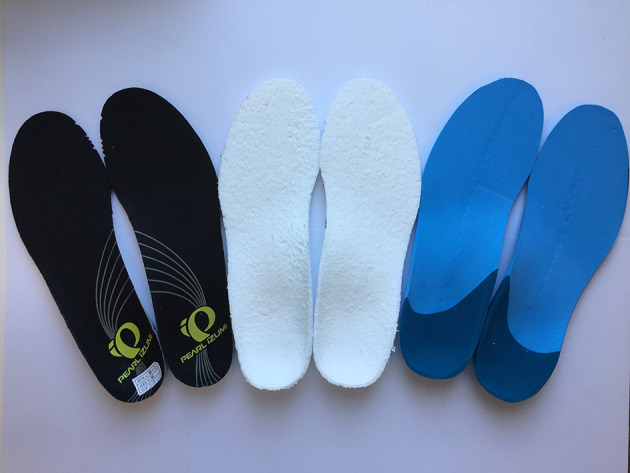
Running Economy (RE) in three different footwear conditions (CON = Shoes only; EVA = Shoes + EVA orthotic; TPU = Shoes + TPU orthotic) over two speeds (“High Speed” = speed at ventilatory threshold and “Low Speed” = 15% below high speed). Note that there was no statistical significance (*P* = 0.11).

Descriptive statistics are presented as mean values ± SD (Tables 1–**3**). Albeit not reaching statistical significance (*P* = 0.11, η^2^ = 0.12), RE on average improved in EVA (+2.1 ± 4.8 and +2.9 ± 4.9%) and TPU (+0.9 ± 5.9 and +0.9 ± 5.3%) compared to CON at LS and HS, respectively (Figure 2). There was a statistically significant main effect of the condition on HR (*P* = 0.04, η^2^ = 0.17) with higher HR and values in CON compared to the other two conditions. No significant speed × condition interaction was found (*P* ≥ 0.28) for any cardio-respiratory variable (Table 1).

**Table 1:** Changes in cardiorespiratory parameters for shoe only (CON), shoe with Ethyl-Vinyl Acetate orthotic (EVA), and shoe with Thermoplastic Poly-Urethane orthotic (TPU) conditions at low and high speeds.

**Parameters**	**Low speed**			**High speed**			**ANOVA *P*-value (η^2^)**		
	**CON**	**EVA**	**TPU**	**CON**	**EVA**	**TPU**	**C**	**S**	**I**
HR (beats.min^−1^)	154.9 ± 14.9	154.4 ± 12.9	152.7 ± 13.6^*^	164.3 ± 13.5	162.9 ± 12.6	161.7 ± 12.9^*^	**0.04** (0.17)	**0.01** (0.38)	0.57 (0.03)
RE (mL.kg^−1^.km^−1^)	191 ± 11	187 ± 14	190 ± 12	190 ± 11	185 ± 14	188 ± 11	0.11 (0.12)	0.11 (0.14)	0.53 (0.04)
RVO_2_ (mL.kg^−1^.min^−1^)	37.60 ± 3.36	36.79 ± 3.57	37.26 ± 3.81	43.73 ± 4.01	42.48 ± 4.51	43.39 ± 4.98	0.11 (0.12)	**<0.001** (0.96)	0.28 (0.07)
VO_2_ (mL.min^−1^)	2808 ± 351	2766 ± 380	2784 ± 390	3265 ± 405	3190 ± 425	3238 ± 448	0.18 (0.10)	**<0.001** (0.96)	0.29 (0.07)
VE (L.min^−1^)	77.9 ± 11.2	77.7 ± 11.4	76.7 ± 10.4	100.9 ± 15.6	100.7 ± 15.2	99.8 ± 15.2	0.60 (0.03)	**<0.001** (0.95)	1.00 (0.00)
BF (breaths.min^−1^)	38.12 ± 6.22	38.88 ± 7.37	38.62 ± 7.15	42.59 ± 6.83	44.91 ± 10.73	44.46 ± 8.26	0.15 (0.11)	**<0.001** (0.65)	0.48 (0.04)
VT (L)	2.08 ± 0.40	2.04 ± 0.48	2.06 ± 0.47	2.39 ± 0.43	2.31 ± 0.57	2.20 ± 0.48	0.21 (0.09)	**<0.001** (0.85)	0.39 (0.05)

Values are mean ± SD. C, S, and I respectively refer to ANOVA main effects of condition, speed and interaction between these two factors with P-value and partial eta-squared (η^2^) in parentheses. HR, Heart rate; RE, Running economy; RVO_2_, Oxygen uptake relative to bodyweight; VO_2_, Absolute oxygen uptake; VE, Minute ventilation; BF, Breathing frequency; VT, Tidal volume. Bold values indicate statistically significant findings.

* *Significantly different from CON (P ≤ 0.05)*.

Almost all examined kinetic variables (except braking loading rate and leg stiffness) increased significantly from LS to HS (*P* ≤ 0.05), irrespective of condition (Table 2). A significant main condition effect was noted for 9 out of 18 variables studied: contact time (*P* = 0.01, η^2^ = 0.28), vertical peak loading rate (*P* ≤ 0.001, η^2^ = 0.32), vertical mean loading rate (*P* = <0.001, η^2^ = 0.52), peak braking force (*P* = 0.03, η^2^ = 0.20), peak propulsive force (*P* = 0.03, η^2^ = 0.23), time peak braking force (*P* ≤ 0.001, η^2^ = 0.55), time peak propulsive force (*P* ≤ 0.001, η^2^ = 0.37), propulsive phase duration (*P* ≤ 0.001, η^2^ = 0.30), propulsive loading rate (*P* = 0.01, η^2^ = 0.25). A significant interaction was found for braking impulse (*P* ≤ 0.05, η^2^ = 0.17) only.

**Table 2:** Changes in running mechanics for shoe only (CON), shoe with Ethyl-Vinyl Acetate orthotic (EVA), and shoe with Thermoplastic Poly-Urethane orthotic (TPU) conditions at low and high speeds.

	**Low speed**			**High speed**			**ANOVA *P*-value (η^2^)**		
	**CON**	**EVA**	**TPU**	**CON**	**EVA**	**TPU**	**C**	**S**	**I**
**Spatiotemporal parameters**									
Contact time (ms)	248 ± 19	248 ± 18	252 ± 20^*^	223 ± 17	225 ± 17	226 ± 17^*^	**0.01** (0.28)	**<0.001** (0.94)	0.08 (0.14)
Flight time (ms)	103 ± 19	104 ± 19	102 ± 17	117 ± 18	116 ± 19	117 ± 17	0.94 (0.00)	**<0.001** (0.89)	0.26 (0.08)
Step frequency (Hz)	2.86 ± 0.15	2.85 ± 0.13	2.83 ± 0.13	2.95 ± 0.15	2.94 ± 0.12	2.92 ± 0.13	0.25 (0.08)	**<0.001** (0.78)	0.92 (0.01)
**Vertical forces**									
Peak vertical force (BW)	2.54 ± 0.28	2.49 ± 0.25	2.49 ± 0.22	2.69 ± 0.27	2.62 ± 0.27	2.65 ± 0.24	0.11 (0.13)	**<0.001** (0.81)	0.54 (0.04)
Vertical peak loading rate (BW.s^−1^)	79.0 ± 17.4	80.2 ± 17.2	73.2 ± 14.9^*^^†^	95.4 ± 19.9	94.7 ± 20.9	89.3 ± 18.4^*^^†^	**<0.001** (0.32)	**<0.001** (0.88)	0.34 (0.07)
Vertical mean loading rate (BW.s^−1^)	51.1 ± 11.3	52.7 ± 11.0	44.4 ± 8.2^*^^†^	62.3 ± 13.8	62.4 ± 14.0	55.0 ± 10.6^*^^†^	**<0.001** (0.52)	**<0.001** (0.88)	0.28 (0.08)
**Horizontal forces**									
Peak braking force (BW)	−0.52 ± 0.11	−0.50 ± 0.11	−0.54 ± 0.10^†^	−0.59 ± 0.11	−0.59 ± 0.13	−0.60 ± 0.11^†^	**0.03** (0.20)	**<0.001** (0.87)	0.18 (0.10)
Peak propulsive force (BW)	0.34 ± 0.06	0.32 ± 0.05	0.32 ± 0.05	0.41 ± 0.06	0.39 ± 0.07	0.39 ± 0.06^*^	**0.03** (0.23)	**<0.001** (0.96)	0.28 (0.07)
Time peak braking force (ms)	61 ± 5	61 ± 7	64 ± 8^*^^†^	57 ± 8	58 ± 7	60 ± 8^*^^†^	**<0.001** (0.55)	**<0.001** (0.44)	0.37 (0.06)
Time peak propulsive force (ms)	184 ± 15	184 ± 15	188 ± 17^*^^†^	167 ± 14	168 ± 14	169 ± 15^*^^†^	**<0.001** (0.37)	**<0.001** (0.87)	0.09 (0.14)
Braking phase duration (ms)	120 ± 11	119 ± 10	121 ± 11	109 ± 9	108 ± 8	110 ± 8	0.13 (0.12)	**<0.001** (0.82)	0.22 (0.09)
Propulsive phase duration (ms)	128 ± 11	129 ± 10	131 ± 12^*^	114 ± 11	116 ± 12	116 ± 12^*^	**<0.001** (0.30)	**<0.001** (0.95)	0.43 (0.05)
Braking impulse (m.s^−1^)	0.24 ± 0.03	0.23 ± 0.03	0.24 ± 0.03	0.26 ± 0.03	0.25 ± 0.03	0.25 ± 0.03	0.21 (0.09)	**<0.001** (0.84)	**0.05** (0.17)
Propulsive impulse (m.s^−1^)	0.25 ± 0.03	0.24 ± 0.03	0.25 ± 0.03	0.27 ± 0.03	0.26 ± 0.03	0.26 ± 0.02	0.15 (0.12)	**<0.001** (0.81)	0.11 (0.14)
Braking loading rate (N.s^−1^)	33.33 ± 14.26	33.38 ± 13.67	32.90 ± 12.91	34.66 ± 15.38	34.56 ± 14.76	34.57 ± 12.39	0.96 (0.00)	0.10 (0.16)	0.94 (0.00)
Propulsive loading rate (N.s^−1^)	24.01 ± 11.41	24.37 ± 12.08	27.59 ± 11.62^†^	31.46 ± 13.25	31.93 ± 14.83	35.11 ± 12.28^†^	**0.01** (0.25)	**<0.001** (0.71)	0.99 (0.00)
**Spring mass characteristics**									
Vertical stiffness (kN.m^−1^)	30.53 ± 44.64	31.50 ± 36.29	30.78 ± 37.80	34.76 ± 45.61	35.53 ± 43.39	35.16 ± 41.60	0.36 (0.06)	**<0.001** (0.84)	0.74 (0.02)
Leg stiffness (kN.m^−1^)	15.21 ± 21.55	15.45 ± 17.70	15.01 ± 20.68	15.76 ± 24.48	15.56 ± 21.65	15.51 ± 21.79	0.39 (0.06)	0.14 (0.13)	0.15 (0.12)

*Values are mean ± SD. C, S, and I respectively refer to ANOVA main effects of condition, speed and interaction between these two factors with P-value and partial eta-squared (η^2^) in parentheses. Bold values indicate statistically significant findings*.

* *Significant different from CON (P ≤ 0.05)*;

† *Significant different from EVA (P ≤ 0.05)*.

**Figure 3:**
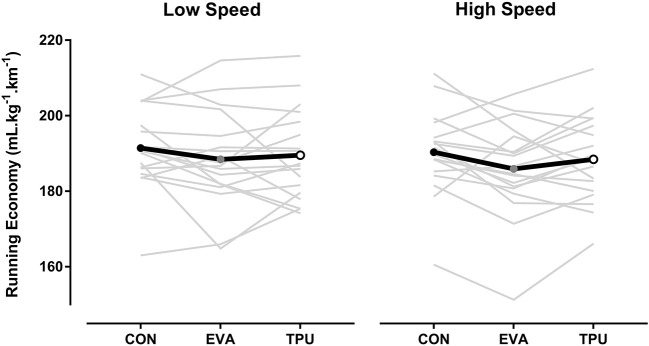
An overview of percentage change for selected mechanical, physiological and comfort parameters when comparing three different conditions (CON-EVA = Shoes only vs. Shoes + EVA orthotic; EVA-TPU = Shoes + EVA orthotic vs. Shoes + TPU orthotic; CON-TPU = Shoes only vs. Shoes + TPU orthotic) over two speeds (“High Speed” = speed at ventilatory threshold and “Low Speed” = 15% below high speed).

Of all the subjective measures (Table 3), only RPE displayed a statistically significant large main effect of speed (*P* < 0.001, η^2^ = 0.77), where higher RPE values were recorded for HS vs. LS. A trend toward improved awareness for arch height comfort was found (*P* = 0.06, η^2^ = 0.19) for both EVA and TPU conditions compared to CON. Also, medio-lateral control (*P* = 0.06, η^2^ = 0.16) was rated as more comfortable for both orthotic conditions compared to CON. Relative average changes (% ± SD) between the three conditions for the most important metabolic, kinetic measures are summarized in Figure 3.

**Table 3:** Changes in rating of perceived exertion (RPE) and comfort parameters for shoe only (CON), shoe with Ethyl-Vinyl Acetate orthotic (EVA), and shoe with Thermoplastic Poly-Urethane orthotic (TPU) conditions at low and high speeds.

**Parameters**	**Low speed**			**High speed**			**ANOVA *P*-value (η^2^)**		
	**CON**	**EVA**	**TPU**	**CON**	**EVA**	**TPU**	**C**	**S**	**I**
RPE	10.4 ± 2.3	10.9 ± 2.6	10.6 ± 2.1	12.7 ± 3.1	13.2 ± 3.1	13.0 ± 3.0	0.14 (0.12)	**<0.001** (0.77)	0.84 (0.01)
Overall comfort	82.5 ± 31.3	93.9 ± 24.4	99.3 ± 24.3	86.0 ± 32.5	93.1 ± 30.6	97.1 ± 25.2	0.21 (0.09)	0.94 (0.00)	0.52 (0.03)
Heel cushioning	82.8 ± 29.9	94.7 ± 24.2	92.5 ± 26.7	82.6 ± 33.3	96.5 ± 22.6	88.6 ± 25.3	0.18 (0.10)	0.68 (0.01)	0.57 (0.03)
Forefoot cushioning	89.8 ± 35.9	96.8 ± 30.9	103.2 ± 23.2	88.2 ± 34.1	96.1 ± 27.9	102.4 ± 23.5	0.30 (0.07)	0.45 (0.04)	0.98 (0.00)
Medio-lateral control	84.4 ± 26.4	100.8 ± 23.9	99.8 ± 25.0	83.1 ± 32.5	97.9 ± 26.4	100.5 ± 25.1	0.06 (0.16)	0.58 (0.02)	0.74 (0.02)
Arch height	77.5 ± 33.0	98.2 ± 32.8	94.3 ± 24.6	74.2 ± 36.1	91.6 ± 33.0	96.4 ± 23.9	0.06 (0.19)	0.13 (0.14)	0.08 (0.16)
Heel cup fit	86.3 ± 27.8	98.8 ± 23.5	87.0 ± 27.5	86.2 ± 28.8	95.3 ± 22.8	88.3 ± 29.1	0.22 (0.09)	0.68 (0.01)	0.50 (0.04)

*RPE was assessed using a 6–20 Borg scale and other comfort parameters were measures using a Visual Analog Scale (0–150 mm); Values are mean ± SD. C, S, and I respectively refer to ANOVA main effects of condition, speed and interaction between these two factors with P-value and partial eta-squared (η^2^) in parentheses. Bold values indicate statistically significant findings*.

## Discussion

The first hypothesis is only partially accepted as EVA improves RE (albeit not significantly) and increases comfort (for cushioning and control, in general and under the heel, arch, and forefoot), in line with more favorable running mechanics (decreasing braking forces) compared to the control condition. The second hypothesis is rejected as the magnitude of these changes was not larger for the higher resilient TPU in comparison to EVA. A unique aspect to this study was also to highlight favorable changes in running mechanics while wearing CFO, yet with material-specific effects. In summary, RE and comfort tended to be improved while wearing either EVA or TPU in reference to CON (with larger effects for the former) but this was not achieved through similar adjustments in running mechanics.

### Running Economy

We reported improved RE (*P* = 0.11, η^2^ = 0.12) mainly when using EVA (+2.1 ± 4.8 and +2.9 ± 4.9%), at HS and LS respectively, compared to CON (*P* = 0.11, η^2^ = 0.12). The positive effect of TPU on RE (+0.9 ± 5.9 and +0.9 ± 5.3%), at HS and LS respectively, is considered as negligible. Similarly to EVA, improved RE of at least ~3% was found by Burke and Papuga (2012) for male participants running at corresponding speeds ranging ~11–15 km.h^−1^ using CFO (Ultrastep®). The larger effect of EVA vs. TPU on RE could be explained by the overall increase of footwear stiffness. With the introduction of CFO, the longitudinal bending stiffness of the footwear (shoe + CFO) would be higher (Levine, 2010), while this effect was probably larger for EVA in reference to TPU (both larger than CON). Reportedly, increases in footwear midsole bending stiffness in the range 6–8% improves RE by 1% (Roy and Stefanyshyn, 2006). Furthermore, lower values observed for RVO_2_ in TPU compared to EVA might be attributed to a higher overall average mass (+34 g) of TPU. This has been described to be detrimental for RE (Hoogkamer et al., 2018). The higher weight and flexibility of TPU compared to EVA possibly dampens the beneficial properties improving RE.

Increased RE corresponded with a reduction up to 4% in HR either while wearing EVA or TPU, at both speeds compared to CON. This result underscores the findings by Kelly et al. (2011), who found a 3% reduction in HR when using CFO made of EVA compared to running in shoes only at a submaximal speed of 10% above the first ventilatory threshold, suggesting CFO may reduce cardiorespiratory load imposed on the runner. However, decreased HR may probably be seen as a consequence rather than a cause of better RE (Barnes and Kilding, 2015).

### Running Mechanics

All examined mechanical variables (except braking loading rate and leg stiffness), for all conditions, changed significantly from LS to HS. Our findings when running at LS and HS (~55 and ~70% of maximal running speed, respectively) are in line with Brughelli et al. (2011) who reported both higher vertical and horizontal forces and shorter contact times with increasing running speeds up to ~65% of maximal velocity.

TPU reduced both mean and peak vertical loading rate by ~12% at both speeds compared to CON and EVA. A lower vertical loading rate and vertical impact force of about ~10% were the only biomechanical differences found between an injured and non-injured group of runners running at ~14 km.h^−1^ (Hreljac et al., 2000). Reductions in vertical loading rate and peak vertical impact forces are suggested to decrease risk of running related injuries (Malisoux et al., 2018). Compared to EVA, TPU used in this study is a softer material dampening initial heel contact. This dampening effect could possibly explain our observations of reductions in peak vertical force and vertical rate of loading.

Another novel aspect is the reporting of horizontal force production as a mechanical variable influenced by different CFO materials. Compared to CON, EVA decreased peak braking forces by ~4% (~0.50 BW) and ~3% (~0.59 BW) and braking impulse by ~3 and ~2% for LS and HS, respectively. For the TPU condition, braking impulse also decreased at HS by ~2% when compared to CON. In contrast, TPU in reference to both CON and EVA produced slightly higher peak braking forces of ~6 and ~3% for LS and HS, respectively. Peak braking forces (~0.27 BW) were identified as the main risk factor for running related injuries in female runners running at moderate intensity of ~9 km.h^−1^ (Napier et al., 2018). Both CFO used in this study had an increased thickness of 8 mm of the heel and forefoot region. The combination of an increased thickness with increased cushioning for TPU possibly facilitates a rearfoot strike pattern during running and is known to increase braking forces (Lieberman et al., 2010). Our findings of acute reduced peak braking forces with EVA are an important observation that might be linked to the reduction of running related injuries. With regard to RE, a reduction of braking impulse will directly result in reduced amount of speed lost during running potentially resulting in a more economical running style (Nummela et al., 2007).

The magnitude of horizontal peak propulsive force was ~4% higher for CON when compared to both EVA and TPU at both HS and LS. However, TPU significantly increased the duration of propulsion by ~2% compared to CON. Also, TPU demonstrated ~18% higher propulsive loading rate values across tested speeds compared to EVA and CON. Worobets et al. (2014) suggested that the limited loss of energy after a loading cycle with TPU could increase resilience and thus improve propulsion. Furthermore, lower vertical impact force, peak medial–lateral force and peak braking force and higher peak propulsive force are typical characteristics of an improved RE (Moore, 2016). Different mechanisms are at play, depending on the materials used for CFO. Where EVA might positively influence RE by reducing the magnitude of braking forces, a longer propulsion duration and increased rate of propulsive force could be a key kinetic modification induced by wearing TPU.

Contact times were slightly longer (1–2%) and associated with slightly decreased step frequency ~1% for both orthotic conditions compared to CON. These observations can also possibly be explained by the effect of increased cushioning with CFO as seen in studies comparing minimalist and traditional footwear (Lohman et al., 2011). In our study, the magnitude of change in spatiotemporal characteristics was probably too narrow to induce measurable changes in RE between conditions.

### Comfort

A trend toward significant improvement of comfort for medio-lateral control (~20%) and arch height (~25%) was found when both EVA and TPU were compared to CON at both speeds. These findings are in line with Burke and Papuga (2012) who reported improved medial-lateral control comfort together with overall comfort, heel cushioning and heel cup fit to correlate with improved RE. Over all measured comfort items, EVA and TPU improved comfort by ~15% for both speeds compared to CON. Molded CFO generally increase comfort that induces functionally relevant changes in running mechanics such as decreased peak vertical force and vertical loading rate (Mundermann et al., 2003b).

### Additional Considerations and Limitations

Because running mechanics may slightly differ between genders and the ground type surfaces, the findings of this study are only valid in the context of male recreational athletes running on a treadmill (Moore, 2016).

Additional mass of footwear is known to have a detrimental influence on RE. For every added 100 g per shoe, the energetic cost of running typically increases by ~1% (Frederick, 1984). In this study, we decided not to match the mass for each shoe/orthotic footwear condition, and by doing so, keeping the interventions clinically relevant by increasing the external validity. The results of this study have shown RE to improve with the different CFO, despite the increased weight of both interventions. TPU and EVA were 14 and 8% heavier compared to CON, respectively. To eliminate the confounding effects of added shoe mass on the energetic cost and running mechanics, future studies should also look to match shoe mass of all conditions to isolate the “true” biomechanical and physiological effects of these two types of CFO.

The approach used in this study to determine the running speeds based on the individual ventilatory threshold determination is a strong methodological point. However, despite this precaution, highly inter-individual responses occurred. An inter-individual variability of 12.5 and 13.3% (CON), 12.4 and 14.0% (EVA) and 13.7 and 13.8% (TPU) was found for RE at LS and HS, respectively. These values were not lower than previously reported for running at set speeds (e.g., 10, 12, 14 km.h^−1^) (Burke and Papuga, 2012). Inter-individual differences, often due to other modifiable and non-modifiable factors (e.g., anthropometrical, biomechanical, physiological, training) may have confounded the findings of this study.

Amount of individualization of the shape of the orthoses in this study was consistent for all participants and no corrective posting was applied. Controlling kinetic and kinematic responses (dose-response) across a group of participants hasn't been investigated previously (Griffiths and Spooner, 2018; Hoogkamer et al., 2018). Our results, with large inter-individual variability, highlight the fact that a “one-fits-all approach” must not be taken when interpreting mechanical and metabolic results. Individual responses, as plotted in Figure 2, highlight the between-subjects variability in RE on a controlled but standardized intervention.

## Conclusion

RE marginally improved (albeit not significantly) when running at two different speeds, while wearing EVA custom foot orthoses compared to CON. The effect of TPU on RE was considered negligible. Comfort improved in the same conditions, while wearing either EVA or TPU in reference to CON, with larger effects for TPU. The footwear condition including EVA reduced braking forces and braking impulse occurring roughly in the first 25% of contact time, whereas TPU was associated with a decreased vertical loading rate and increased rate of force production during the propulsive phase. Male recreational athletes returning to competition can keep wearing their EVA orthoses.

## Data Availability Statement

The datasets generated for this study are available on request to the corresponding author.

## Ethics Statement

All subjects gave written informed consent in accordance with the Declaration of Helsinki and this study was carried out with the recommendations and approval of the Anti-Doping Laboratory Ethics Committee in Qatar (IRB Application Number 2017000201).

## Author Contributions

KV and OG contributed conception, design of the study, and collected all data. JR analyzed, datamined, and organized the kinematic database. KV performed the statistical analysis and wrote the first draft of the manuscript. All authors wrote sections of the manuscript and contributed to manuscript revision, read, and approved the submitted version.

### Conflict of Interest

The authors declare that the research was conducted in the absence of any commercial or financial relationships that could be construed as a potential conflict of interest.

## References

[B1] Arellano C. J. Kram R. (2014). Partitioning the metabolic cost of human running: a task-by-task approach. Integr. Comp. Biol. 54, 1084–1098. 10.1093/icb/icu033 24838747 PMC4296200

[B2] Barnes K. R. Kilding A. E. (2015). Running economy: measurement, norms, and determining factors. Sports Med. Open 1:8. 10.1186/s40798-015-0007-y 27747844 PMC4555089

[B3] Borg G. A. (1982). Psychophysical bases of perceived exertion. Med. Sci. Sports Exerc. 14, 377–381. 7154893

[B4] Brughelli M. Cronin J. Chaouachi A. (2011). Effects of running velocity on running kinetics and kinematics. J. Strength Cond. Res. 25, 933–939. 10.1519/JSC.0b013e3181c64308 20703170

[B5] Burke J. R. Papuga M. O. (2012). Effects of foot orthotics on running economy: methodological considerations. J. Man. Manip. Ther. 35, 327–336. 10.1016/j.jmpt.2012.04.001 22632593

[B6] Cavagna G. A. (1975). Force platforms as ergometers. J. Appl. Physiol. 39, 174–179. 10.1152/jappl.1975.39.1.174 1150585

[B7] Chang Y. H. Kram R. (1999). Metabolic cost of generating horizontal forces during human running. J. Appl. Physiol. 86, 1657–1662. 10.1152/jappl.1999.86.5.1657 10233132

[B8] Cohen J. (1988). Statistical Power Analysis for the Behavioral Sciences. Hillsdale, MI: L. Erlbaum Associates.

[B9] Davis J. A. (1985). Anaerobic threshold: review of the concept and directions for future research. Med. Sci. Sports Exerc. 17, 6–21. 3884961

[B10] De Wit B. De Clercq D. Aerts P. (2000). Biomechanical analysis of the stance phase during barefoot and shod running. J. Biomech. 33, 269–278. 10.1016/s0021-9290(99)00192-x 10673110

[B11] Frederick E. C. (1984). Physiological and ergonomics factors in running shoe design. Appl. Ergon. 15, 281–287. 10.1016/0003-6870(84)90199-6 15676526

[B12] Fuller J. T. Bellenger C. R. Thewlis D. Tsiros M. D. Buckley J. D. (2015). The effect of footwear on running performance and running economy in distance runners. Sports Med. 45, 411–422. 10.1007/s40279-014-0283-6 25404508

[B13] Girard O. Brocherie F. Morin J. B. Racinais S. Millet G. P. Periard J. D. (2017). Mechanical alterations associated with repeated treadmill sprinting under heat stress. PLoS ONE 12:e0170679. 10.1371/journal.pone.0170679 28146582 PMC5287483

[B14] Griffiths I. B. Spooner S. K. (2018). Foot orthoses research: identifying limitations to improve translation to clinical knowledge and practice. Br. J. Sports Med. 52:350. 10.1136/bjsports-2016-096269 27789429

[B15] Hayes J. Smith L. Santopietro F. (1983). The effects of orthotics on the aerobic demands of running. Med. Sci. Sports Exerc. 15:169.

[B16] Hennig E. M. Valiant G. A. Liu Q. (1996). Biomechanical Variables and the perception of cushioning for running in various types of footwear. J. Appl. Biomech. 12, 143–150. 10.1123/jab.12.2.143

[B17] Hoogkamer W. Kipp S. Frank J. H. Farina E. M. Luo G. Kram R. (2018). A comparison of the energetic cost of running in marathon racing shoes. Sports Med. 48, 1009–1019. 10.1007/s40279-017-0811-2 29143929 PMC5856879

[B18] Hreljac A. Marshall R. N. Hume P. A. (2000). Evaluation of lower extremity overuse injury potential in runners. Med. Sci. Sports Exerc. 32, 1635–1641. 10.1097/00005768-200009000-00018 10994917

[B19] Karp J. R. (2008). An in-depth look at running economy. Track Coach 182, 5801–5806.

[B20] Kelly L. A. Girard O. Racinais S. (2011). Effect of orthoses on changes in neuromuscular control and aerobic cost of a 1-h run. Med. Sci. Sports Exerc. 43, 2335–2343. 10.1249/MSS.0b013e31822037ca 21552159

[B21] Kirby K. A. (2017). Evolution of foot orthoses in sports, in Athletic Footwear and Orthoses in Sports Medicine, eds Werd M. B. Knight E. L. Langer P. R. (Cham: Springer International Publishing), 19–40.

[B22] Kirby K. A. Werd M. B. (2014). The evolution of foot orthoses in sports-part I. Podiatry Manage. 119–123.

[B23] Levine D. (2010). Athletic shoe evaluation, in Athletic Footwear and Orthoses in Sports Medicine, eds Werd M. B. Knight E. L. (New York, NY: Springer New York), 55–62.

[B24] Lieberman D. E. Venkadesan M. Werbel W. A. Daoud A. I. D'Andrea S. Davis I. S.. (2010). Foot strike patterns and collision forces in habitually barefoot versus shod runners. Nature 463, 531–535. 10.1038/nature08723 20111000

[B25] Lohman E. B. III. Balan Sackiriyas K. S. Swen R. W. (2011). A comparison of the spatiotemporal parameters, kinematics, and biomechanics between shod, unshod, and minimally supported running as compared to walking. Phys. Ther. Sport 12, 151–163. 10.1016/j.ptsp.2011.09.004 22085708

[B26] MacLean C. L. Davis I. S. Hamill J. (2008). Short- and long-term influences of a custom foot orthotic intervention on lower extremity dynamics. Clin. J. Sport Med. 18, 338–343. 10.1097/MJT.0b013e31815fa75a 18614885

[B27] Malisoux L. Gette P. Meyer C. Urhausen A. Theisen D. (2018). Factors influencing loading rate of vertical impact forces in running. Sports Orthop. Traumatol. 34, 215–216. 10.1016/j.orthtr.2018.03.097

[B28] McCormick C. J. Bonanno D. R. Landorf K. B. (2013). The effect of customised and sham foot orthoses on plantar pressures. J. Foot Ankle Res. 6:19. 10.1186/1757-1146-6-19 23680496 PMC3663766

[B29] McPoil T. G. Vicenzino B. Cornwall M. W. (2011). Effect of foot orthoses contour on pain perception in individuals with patellofemoral pain. J. Am. Podiatry Assoc. 101, 7–16. 10.7547/1010007 21242465

[B30] Milani T. L. Schnabel G. Hennig E. M. (1995). Rearfoot motion and pressure distribution patterns during running in shoes with varus and valgus wedges. J. Appl. Biomech. 11, 177–187. 10.1123/jab.11.2.177

[B31] Moore I. S. (2016). Is there an economical running technique? A review of modifiable biomechanical factors affecting running economy. Sports Med. 46, 793–807. 10.1007/s40279-016-0474-4 26816209 PMC4887549

[B32] Morin J. B. Dalleau G. Kyrolainen H. Jeannin T. Belli A. (2005). A simple method for measuring stiffness during running. J. Appl. Biomech. 21, 167–180. 10.1123/jab.21.2.167 16082017

[B33] Mundermann A. Nigg B. M. Humble R. N. Stefanyshyn D. J. (2003a). Foot orthotics affect lower extremity kinematics and kinetics during running. Clin. Biomech. 18, 254–262. 10.1016/s0268-0033(02)00186-9 12620789

[B34] Mundermann A. Nigg B. M. Humble R. N. Stefanyshyn D. J. (2003b). Orthotic comfort is related to kinematics, kinetics, and EMG in recreational runners. Med. Sci. Sports Exerc. 35, 1710–1719. 10.1249/01.MSS.0000089352.47259.CA 14523309

[B35] Mundermann A. Nigg B. M. Stefanyshyn D. J. Humble R. N. (2002). Development of a reliable method to assess footwear comfort during running. Gait Posture 16, 38–45. 10.1016/S0966-6362(01)00197-7 12127185

[B36] Napier C. MacLean C. L. Maurer J. Taunton J. E. Hunt M. A. (2018). Kinetic risk factors of running-related injuries in female recreational runners. Scand. J. Med. Sci. Sports 28, 2164–2172. 10.1111/sms.13228 29846979

[B37] Nummela A. Keranen T. Mikkelsson L. O. (2007). Factors related to top running speed and economy. Int. J. Sports Med. 28, 655–661. 10.1055/s-2007-964896 17549657

[B38] Redmond A. C. Crosbie J. Ouvrier R. A. (2006). Development and validation of a novel rating system for scoring standing foot posture: the foot posture index. Clin. Biomech. 21, 89–98. 10.1016/j.clinbiomech.2005.08.002 16182419

[B39] Roy J. P. Stefanyshyn D. J. (2006). Shoe midsole longitudinal bending stiffness and running economy, joint energy, and EMG. Med. Sci. Sports Exerc. 38, 562–569. 10.1249/01.mss.0000193562.22001.e8 16540846

[B40] Shaw A. J. Ingham S. A. Fudge B. W. Folland J. P. (2013). The reliability of running economy expressed as oxygen cost and energy cost in trained distance runners. Appl. Physiol. Nutr. Metab. 38, 1268–1272. 10.1139/apnm-2013-0055 24195628

[B41] Sinclair J. McGrath R. Brook O. Taylor P. J. Dillon S. (2016). Influence of footwear designed to boost energy return on running economy in comparison to a conventional running shoe. J. Sports Sci. 34, 1094–1098. 10.1080/02640414.2015.1088961 26367197

[B42] Worobets J. Wannop J. W. Tomaras E. Stefanyshyn D. (2014). Softer and more resilient running shoe cushioning properties enhance running economy. Footwear Sci. 6, 147–153. 10.1080/19424280.2014.918184

[B43] Zeintl C. (2018). Infinergy® - BASF - We Create Chemistry. Available online at: https://www.plasticsportal.net/wa/plasticsEU/portal/show/common/content/campaigns/infinergy/english/index.html (accessed July 4, 2018).

